# Cell recognition based on topological sparse coding for microscopy imaging of focused ultrasound treatment

**DOI:** 10.1186/s12880-015-0087-7

**Published:** 2015-10-24

**Authors:** Zhenyou Wang, Jiang Zhu, Yanmei Xue, Changxiu Song, Ning Bi

**Affiliations:** School of Mathematics and Computational Science, Sun Yat-sen University, Guangzhou, P. R. China; Faculty of Applied Mathematics, Guangdong University of Technology, Guangzhou, P.R. China; The School of Mathematics & Statistics, Nanjing University of Information Science Technology, Nanjing, Jiangsu P.R. China; Department of Ultrasound, Sir Run Shaw Hospital, College of Medicine ZheJiang University, Hangzhou, P.R. China

**Keywords:** Topological continuity characteristics, Sparse coding, Focused ultrasound, Microscopy imaging

## Abstract

**Background:**

Ultrasound is considered a reliable, widely available, non-invasive, and inexpensive imaging technique for assessing and detecting the development phases of cancer; both *in vivo* and *ex vivo*, and for understanding the effects on cell cycle and viability after ultrasound treatment.

**Methods:**

Based on the topological continuity characteristics, and that adjacent points or areas represent similar features, we propose a topological penalized convex objective function of sparse coding, to recognize similar cell phases.

**Results:**

This method introduces new features using a deep learning method of sparse coding with topological continuity characteristics. Large-scale comparison tests demonstrate that the RAW can outperform SIFT GIST and HoG as the input features with this method, achieving higher sensitivity, specificity, F1 score, and accuracy.

**Conclusions:**

Experimental results show that the proposed topological sparse coding technique is valid and effective for extracting new features, and the proposed system was effective for cell recognition of microscopy images of theMDA-MB-231 cell line. This method allows features from sparse coding learning methods to have topological continuity characteristics, and the RAW features are more applicable for the deep learning of the topological sparse coding method than SIFT GIST and HoG.

## Background

Knowledge of cell viability, the cytoskeletal system, cell morphology, cell migration, tumor cell inhibition rate, and cell cycle (interphase, prophase, metaphase, and anaphase) are important for understanding various diseases, notably cancer [1, 2]. Changes in the cell cycle before and after drug treatment are useful for effective drug discovery research [3]. Critical to such measurements is the accurate recognition of mitotic cells in a cell culture via automated image analysis. Hundreds of thousands of living cells are recorded in time-lapse phase-contrast microscopy images or microscopy video for research studies in cancer biology and biomaterials engineering [4].

Breast cancer has accounted for approximately 30 % of all female cancers diagnosed in the European Union, and is the leading cause of female cancer deaths. Over 85,000 women (many in their reproductive and economically productive years) have succumbed to the disease [5, 6]. Traditional methods for cell recognition in microscopy images still have several limitations, although much progress has been made. However, some processes of irregular appearance, such as cell death, cytoskeletal and cell morphology changes, cell migration, and cell cycle are difficult to follow. Learning the complex relationships of the multiple states induces high computational complexity and drives the system far from the goal of real-time recognition. Hence, because of the complexity of cell behaviors and morphological variance, existing automatic systems remain limited when dealing with large volumes of time-lapse microscopy images [7, 8]. At the same time, sparse modeling is one of the most successful recent signal processing paradigms, and topological features are better represented as the adjacent and similar points or areas have been extracted from the features of all points or areas. Topology of the topology sparse coding mainly simulates and describes a phenomenon and characteristics so that the adjacent neurons of the human cerebral cortex can extract a similar feature. Topological maps have features wherein adjacent points or areas correspond to adjacent points or areas in feature space, and adjacent points or areas tend to respond to similar features. Feature preference varies smoothly across the cortex, that is to say, adjacent points or areas represent similar features. These are the topological continuity properties [9].

Aapo Hyvärinen and Patrik O. Hoyer [10] have shown that this single principle of sparseness can also lead to emergence of topography and complex cell properties. Rodolphe Jenatton [11] considered an extension of this framework where the atoms are further assumed to be embedded in a tree. This is achieved using a recently introduced tree-structured sparse regularization norm, which has proven useful in several applications. The procedure has a complexity linear, or close to linear, in the number of atoms, and allows the use of accelerated gradient techniques to solve the tree-structured sparse approximation problem at the same computational cost as traditional ones, using L_1_ norm. However, this method has no continuity properties for the same cell phase for different cells, and the gradient method applied here is not normal, because the L_1_ norm of the non-differentiable at point zero.

In this paper, we propose a recognition method based on topological sparse coding. First, cell shape information is obtained using binarization [12]. The detected cells are then segmented via a seeded watershed algorithm [13]. After segmentation, a favorite matching plus local tree matching approach is used to track the dynamic behaviors of cell nuclei [14]. After obtaining segmented nuclei ROIs (regions of interest), each cell is represented by a region feature. Based on these results, we have designed a topological penalized convex objective function to induce sparsity and consistency constraints for dictionary learning and sparse decomposition. Finally, a support vector machine (SVM) classifier is utilized for model learning and prediction. This approach can be used to analyze the behavior of cells as extracted from a time-lapse microscopy video. For instance, we have used this analysis to identify cell phase and cell cycle progress in MDA-MB-231 cells.

## Methods

The MDA-MB-231 cell line from the American Type Culture Collection (ATCC), frozen by the Cornell University Weill Medical College of The Methodist Hospital Research Institute was used. All experimental research reported in this manuscript consisted of *in vitro* experiments.

**Fig. 1:**
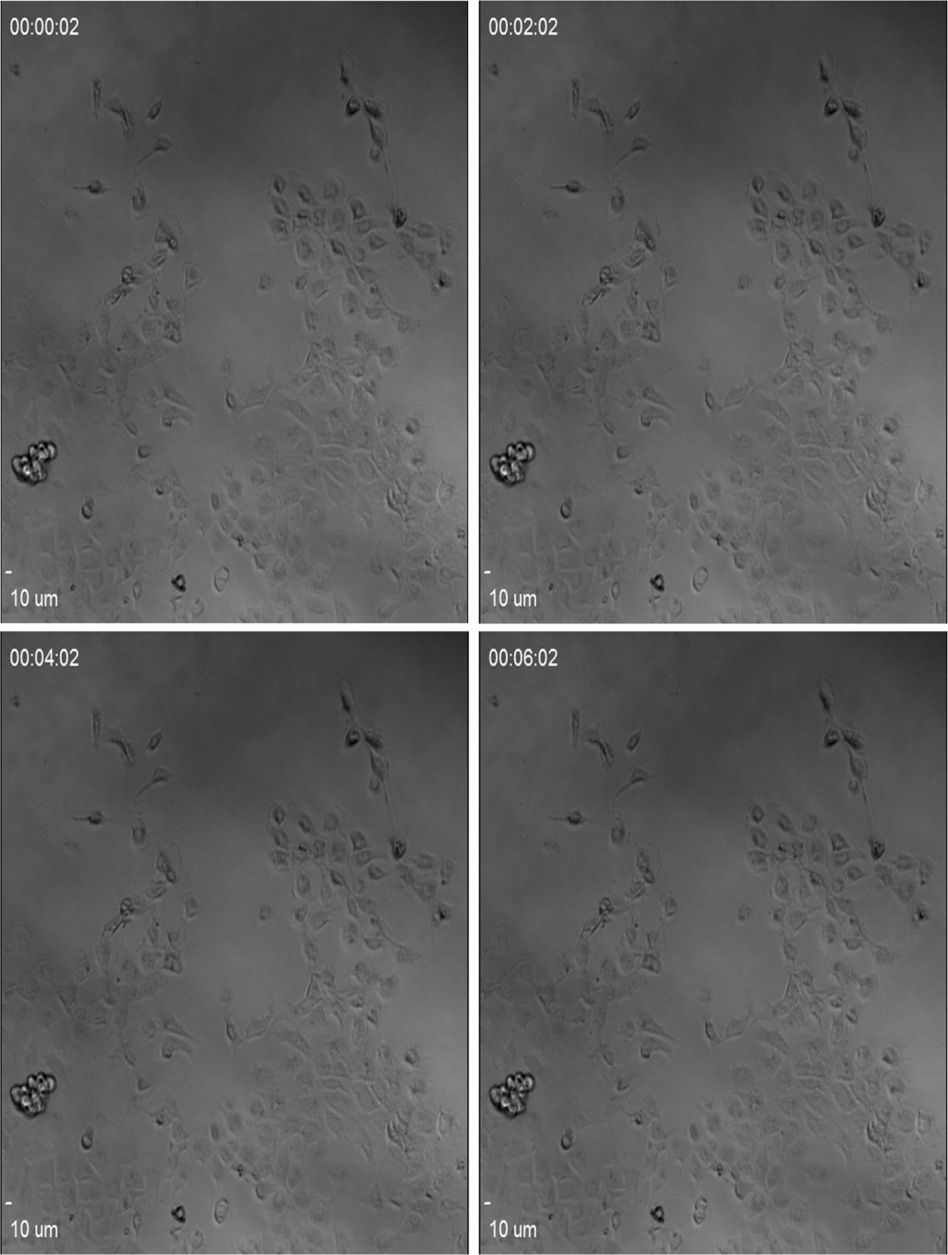
Microscopy image of MDA-MB-231 cell lines. The 4 continuous images of the position 1 with sound of pressure 1Mpa for the each time 00:00:02,00:02:02,00:04:02, 00:06:02

Images were acquired every 2 min for 12 h and 22 min, giving a total of 373 images per hole that were then exported from Simple PCI as 16 bit uncompressed TIFF files to 8 GB network attached storage (NAS) arrays for processing. Figure 1 shows the microscopy images the MDA-MB-231 cells.

First, cell shape information is obtained by binarization. The detected cells are then segmented via a seeded watershed algorithm. After segmentation, a favorite matching plus local tree matching approach is used to track the dynamic behaviors of the cell nuclei.

A pixel-wise intensity feature (Raw) represents the global intensity distribution of one image and implicitly contains its appearance characteristics. Histogram of Oriented Gradients (HoG) [15], Generalized Search Tree (GIST) [16], and Scale Invariant Feature Transform (SIFT) [17] are features that are widely used to represent shape characteristics, local structural information, and local visual saliency, respectively. For comparison, we extracted the pixel-wise intensity feature and three representative visual features from every nuclei [18]. After obtaining feature vectors that include information on shape and texture, they are input into deep learning process. After obtaining segmented nuclei ROIs (regions of interest), each cell is represented by a feature vector including 54 elements for the RAW, converting each candidate into a feature vector that implicitly represents the characteristics of the mitotic cell [19]. In this paper, we input the feature vectors into a topological sparse coding process.

Given a new sample and its feature $\mathrm{x}\;\left(\mathrm{x}\in {\mathrm{R}}^{{}^{\mathrm{d}}}\right)$, The value of “d” is the vector x_i_ of the matrix x has “d” elements. The goal of sparse coding is to decompose it over a dictionary A, such that x = As + r, a set of N data points × in the Euclidean space R^d^ is written as the approximate product of a d × k dictionary A and k × N coefficients s, r is the residual. Least squares estimation (LSE), a similar model fitting procedure, is usually formulated as a minimization of the residual sum of squares to get an optimal coefficient s. However, LSE often poorly preserves both low prediction error and the high sparsity of coefficients [20]. Therefore, penalization techniques have been widely researched to improve on it. Considering the constraints of sparsity and consistency for decomposition, we designed a topological objective function for the system as follows:

1 $$ \mathrm{J}\;\left(\mathrm{A},\;\mathrm{s}\right)={\left\Vert \mathrm{A}\mathrm{s}\;\hbox{-}\;\mathrm{x}\right\Vert}_2^2+\uplambda \kern0.48em {\displaystyle {\sum}_{\mathrm{i},\;\mathrm{all}\;\mathrm{group}}\sqrt{{\mathrm{Vs}}_{\mathrm{i}}{{\mathrm{s}}_{\mathrm{i}}}^{\mathrm{T}}}}. $$

where ‖s ‖_2_^2^ = ∑_i_‖ s_i_ ‖_2_^2^, the s_i_ is the i-th row vector of the coefficient s, where V is the grouping matrix, where the group contains all of the elements of the learning set. For example, if V is 3*3 grouping matrix method, and one group begins from the 1-st row and 2-nd column, so the $\sqrt{{\mathrm{Vs}}_{\mathrm{i}}{{\mathrm{s}}_{\mathrm{i}}}^{\mathrm{T}}}=\sqrt{{\mathrm{s}}_{12}^2+{\mathrm{s}}_{13}^2+{\mathrm{s}}_{14}^2+{\mathrm{s}}_{22}^2+{\mathrm{s}}_{23}^2+{\mathrm{s}}_{24}^2+{\mathrm{s}}_{32}^2+{\mathrm{s}}_{33}^2+{\mathrm{s}}_{34}^2}.$ Small mini-batches, that is to say, we have taken learning sets into several small learning sets. Because the s_i_ is the i-th row vector of the coefficient s, the s_i_^T^ is the column vector, V is the grouping matrix, so V{s_i}{s_i}^t is a value, and then the $\sqrt{{\mathrm{Vs}}_{\mathrm{i}}{{\mathrm{s}}_{\mathrm{i}}}^{\mathrm{T}}}$ in the J(A,s) is the ‖s ‖_1_, and we have reserved the main values of the vector used by L1 norm. So the objective functions are described as “topological penalized.” The objective function in Equation (1) consists of two parts, the first term penalizes the sum-of-squares difference between the reconstructed and original sample; the second term is the sparsity penalty term that is used to guarantee the sparsity of the feature set through a smaller coefficient λ values. The gradient method is not valid at point zero because L_1_ norm is not differentiable at point zero. We then use $\sqrt{{\mathrm{Vs}}_{\mathrm{i}}{{\mathrm{s}}_{\mathrm{i}}}^{\mathrm{T}}+\upvarepsilon}$ that defines a smoothed topographic L_1_ sparsity penalty on *s* in sparse coding instead of $\sqrt{{\mathrm{Vs}}_{\mathrm{i}}{{\mathrm{s}}_{\mathrm{i}}}^{\mathrm{T}}}$ on the L_1_ norm smoothing, where ε is a constant.

J (A, s) is not convex if J (A, s) only includes the first term and second term, but given A, the minimum of J(A,s) to solve s is convex [21, 22]; similarly, given s, minimizing J(A,s) to solved A is also convex, so we add the third term, the weighted decay term with weighted decay coefficients γ into the J (A, s) and then the optimization computation may use the gradient techniques. In order to achieve the following purposes: only a few coefficients values of matrix A are far greater than 0, nor that most coefficients are greater than 0. In order to solve this problem, we can make a constraint on the values of s, C is a constant.

2 $$ \begin{array}{l} \min\;\mathrm{J}\;\left(\mathrm{A},\;\mathrm{s}\right)={\left\Vert \mathrm{A}\mathrm{s}\;\hbox{-}\;\mathrm{x}\;\right\Vert}_2^2+\uplambda \kern0.24em {\displaystyle {\sum}_{\mathrm{i},\;\mathrm{all}\;\mathrm{group}}\sqrt{{\mathrm{Vs}}_{\mathrm{i}}{{\mathrm{s}}_{\mathrm{i}}}^{\mathrm{T}}+\upvarepsilon}+\upgamma\;{\left\Vert\;\mathrm{A}\;\right\Vert}_2^2}\\ {}\mathrm{s}.\mathrm{t}\;{\left\Vert\;\mathrm{s}\;\right\Vert}_2^2\le \mathrm{C}\end{array} $$

Assuming there are enough mitotic cell training samples such that dictionary Α is over-complete, it is clear that a new mitotic cell image can be faithfully represented by a linear combination of mitotic bases contained in A. However, in reality, it is impossible to enumerate all mitotic cases for the training set. Under the sparse coding scheme, each candidate × is represented as a linear combination of bases in matrix Α by coefficient s. Therefore, s explicitly reflects the relationship between $\mathrm{x}$. d the bases and it can be utilized as the characteristic representation for classification. If the iterative algorithm is executed on large data sets, iteration should take a long time and this algorithm also takes a long time to reach convergence results. So we choose to run the algorithm on a mini-block, so that we can improve the speed of iteration and improve the convergence speed.

To optimize the cost function, we follow these steps:

1. Randomly initialize the A function
2. Repeat the following steps until convergence:
3. Randomly select small mini-batches of the learning sets.
4. $\mathrm{s}\leftarrow {\mathrm{A}}^{\mathrm{T}}\mathrm{x},\;{\mathrm{s}}_{\mathrm{r},\mathrm{c}}\leftarrow \frac{{\mathrm{s}}_{\mathrm{r},\mathrm{c}}}{\left\Vert {\mathrm{A}}_{\mathrm{c}}\right\Vert }$ where s_r,c_ is the r-th feature of the c-th sample and ${\mathrm{A}}_{\mathrm{c}}$ is the c-th base vector of matrix A (This is an iteration, all have taken place in the mini-batches).
5. Calculate **s** by minimizing J (A, s) according to equation 2 with gradient techniques (we have calculated the cost function J using gradient descent method (deflector for extreme values of the function), and we have obtained the s used stable point when we have fixed the A).
6. Obtain A such that J (A, s) is minimized according to s with gradient techniques (We have calculated the cost function J using gradient descent method (deflector for extreme values of the function). We have obtained the A used stable point when we have fixed the s).

After these steps, we obtain the topological characteristic feature vectors from the same cell phase. These feature vectors may be classified with the SVM classifier. The following diagram is the overview diagram of the algorithm.

The basic procedure for applying SVM to cell phase recognition is as follows [23]. First, the input vectors are linearly or non-linearly mapped into a feature space (possibly with a higher dimension) by selecting a relevant kernel function. In this paper, the kernel function $\mathrm{k}\;\left(\mathrm{x},\;\mathrm{x}^{\prime}\right)=\frac{{\left\Vert \mathrm{x}\;\hbox{-}\;\mathrm{x}\prime \right\Vert}^2}{\updelta}$ is used. Then, within this feature space, an optimized linear division is sought by constructing a hyper-plane that separates the samples into four classes (interphase, prophase, metaphase, and anaphase) with the least errors and maximal margin. The SVM training process always seeks a globally optimized solution and avoids over fitting [23], hence, SVM has the ability to deal with a large number of features.

## Results and discussion

We took the first 240 images of the data set as the learning set and the other 133 images for the test set. This generated a learning set consisting of 19521 nuclei and test set consisting of 10881 nuclei, where we were mainly concerned with the cell cycle phase (interphase, prophase, metaphase, and anaphase). After computation on matrix A with gradient techniques, the dimensionality of $\mathrm{x}$. the experiments are 54 × 19521, the dimensionality of Α in the experiments are 54 × 121, the dimensionality of $\mathrm{s}$. the experiments are 121 × 19,521.

To demonstrate the superiority of the proposed method for mitotic cell recognition, we evaluated the sensitivity and specificity of our experimental results. We compared the performances on the same test set for mitotic cell recognition. Let TP, TN, FP, and FN stand for the number of true positive, true negative, false positive, and false negative samples, respectively, after the completion of cell phase identification. Sensitivity is defined as: (TP/(TP + FN)), and is a statistical measure of how well-classified the positive cells are. Specificity reflects the ability to identify negative cells correctly and is defined as (TN/(TN + FP)). Precision is (TP/(TP + FP)), accuracy is ((TP + TN)/(TP + FN + FP + TN)), and the F1 score ((2 ×∁ precision × sensitivity)/(precision + sensitivity)) represents the overall performance of both. These are commonly-used quantitative metrics to evaluate the performance of mitotic cell recognition. λ and γ are again trade-off parameters controlling the balance between the reconstruction quality and sparsity [24, 25], when comparing the performance of different dictionary learning strategies with four visual features and different configurations, λ and γ were set to 0.1 [26, 27] and C is set to 1.

**Fig. 2:**
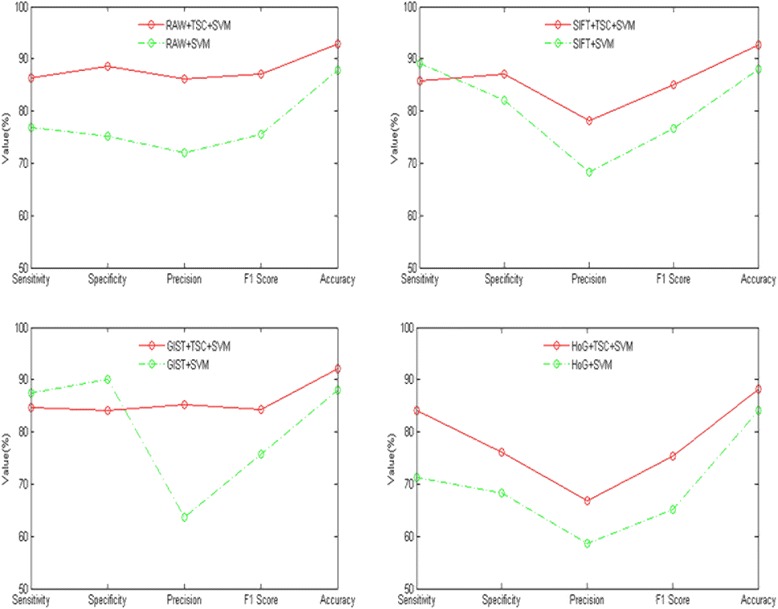
Comparison with topological sparse coding deep learning or not. Red lines represents the algorithm have the deep learning step of topology sparse coding, green lines represent the algorithm don't have the deep learning step of topology sparse coding. From left to right of the first row are: RAW as input characteristics, SIFT as input characteristics. From left to right of the second row are: GIST as input characteristics, HoG as input characteristics

From Fig. 2, for each index of the RAW and HoG features, including sensitivity specificity precision F1 score accuracy, the classification performance with topological sparse coding deep learning was better than with none.

**Fig. 3:**
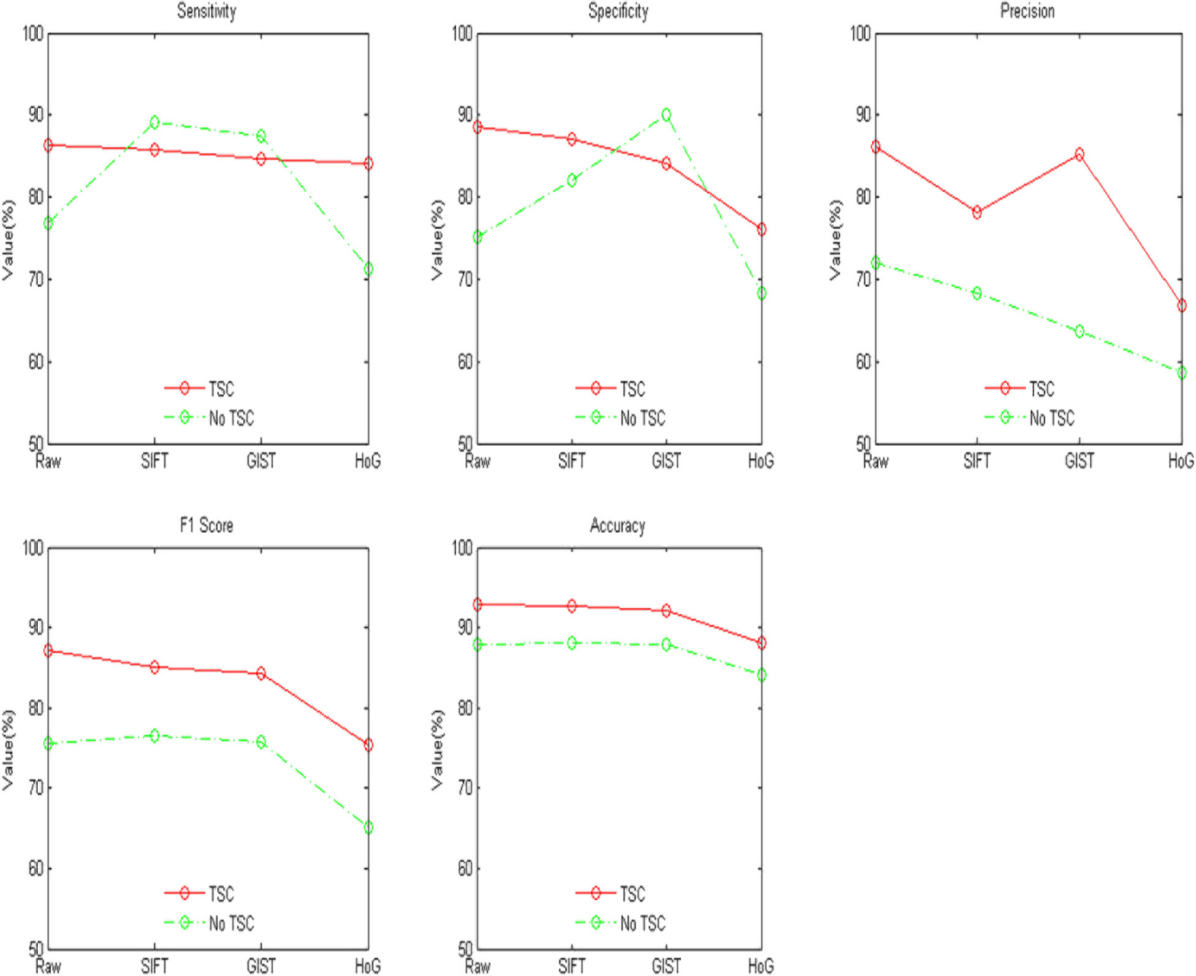
Comparison with topological sparse coding deep learning or not. Red lines represents the algorithm has the deep learning step of topology sparse coding, green lines represent the algorithm does not have the deep learning step of topology sparse coding. From left to right of the first row are: sensitivity, specificity, precision. From left to right of the second row are:, F1 score, accuracy

From Fig. 3, for precision F1 score and accuracy indexes of the RAW SIFT GIST and HoG features, the classification performance with topological sparse coding deep learning was better than with none.

**Fig. 4:**
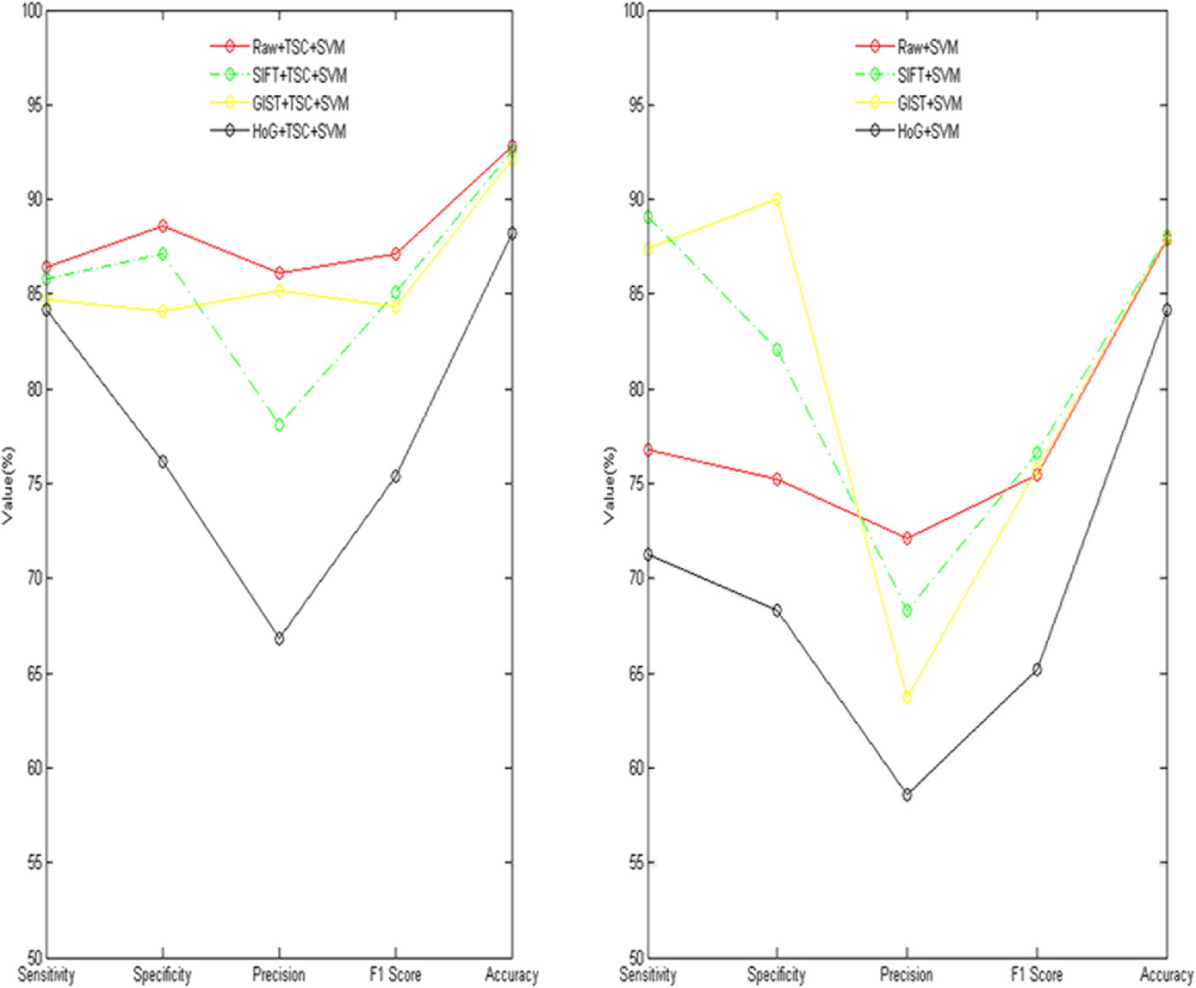
Comparison of each type input characteristics.The left figure is algorithm have the deep learning step of topology sparse coding; and the right figure is algorithm don't have the deep learning step of topology sparse coding

From left part of Fig. 4, for each index including sensitivity specificity precision F1 score accuracy, under the condition of topological sparse coding deep learning, the classification performance used RAW feature as input feature is better than SIFT GIST and HoG features. The right part of the Fig. 4 has the same results; that is the classification performance used RAW feature as input feature is better than SIFT GIST and HoG features with no deep learning. The HoG feature performs poorer than RAW SIFT and GIST. It was thought that HoG would be the least accurate because it is not very suitable for deformable object representation in this case.

The extracting features method of topological sparse coding with topological continuity characteristics is feasible and effective for deep learning. The index of RAW with deep learning is higher than the others, implying that a pixel-wise intensity feature (RAW) represents the global intensity distribution of one image and implicitly contains its appearance characteristics. In addition, the RAW features are more applicable for deep learning of the topological sparse coding method than the SIFT, GIST, and HoG features.

Finally, we have compared our results with Mairal et al.’s unsupervised and supervised approaches [13], and sparse the coding for reWL1 [28]. The best results of these approaches are shown in Table 1. Our error rate is significantly better compared to theirs. However, it should be noted that we have used the RAW feature in all our experiments.

**Table 1:** Error rate for different approaches

	Mairal et al. [13]	Mairal et al. [13]	reWL1	RAW+TSC+SVM
approaches	(unsupervised)	(supervised)	[28] (WL1)	approache in the paper
Error rate	12.02 %	7.93 %	9.71 %	7.21 %

## Conclusions

In this paper, we proposed a topological penalized convex objective function of sparse coding for the recognition of cell cycles, based on the fact that topology of the topology sparse coding mainly describes a phenomenon and characteristics that the adjacent neurons of the human cerebral cortex can extract a similar feature. This method has made the new features from the deep learning methods of sparse coding to have topological continuity characteristics. Large-scale comparison tests demonstrate that the RAW can outperform SIFT GIST and HoG, achieving higher sensitivity, specificity, F1 score, and accuracy. That is to say, the proposed topological sparse coding technique is valid and effective for the extracting of new features, and the RAW features are more applicable for the deep learning of the topological sparse coding method than SIFT GIST and HoG.
